# Serum Antibodies Against the Oncogenic Merkel Cell Polyomavirus Detected by an Innovative Immunological Assay With Mimotopes in Healthy Subjects

**DOI:** 10.3389/fimmu.2021.676627

**Published:** 2021-06-08

**Authors:** Chiara Mazziotta, Carmen Lanzillotti, Elena Torreggiani, Lucia Oton-Gonzalez, Maria Rosa Iaquinta, Elisa Mazzoni, Pauline Gaboriaud, Antoine Touzé, Ettore Silvagni, Marcello Govoni, Fernanda Martini, Mauro Tognon, John Charles Rotondo

**Affiliations:** ^1^ Department of Medical Sciences, University of Ferrara, Ferrara, Italy; ^2^ ISP “Biologie des infections à polyomavirus” Team, UMR INRA 1282, University of Tours, Tours, France

**Keywords:** indirect ELISA, Merkel cell polyomavirus, antibodies, serology, IgGs, MCPyV, polyomavirus, Merkel cell carcinoma

## Abstract

Merkel cell polyomavirus (MCPyV), a small DNA tumor virus, has been detected in Merkel cell carcinoma (MCC) and in normal tissues. Since MCPyV infection occurs in both MCC-affected patients and healthy subjects (HS), innovative immunoassays for detecting antibodies (abs) against MCPyV are required. Herein, sera from HS were analyzed with a novel indirect ELISA using two synthetic peptides mimicking MCPyV capsid protein epitopes of VP1 and VP2. Synthetic peptides were designed to recognize IgGs against MCPyV VP mimotopes using a computer-assisted approach. The assay was set up evaluating its performance in detecting IgGs anti-MCPyV on MCPyV-positive (n=65) and -negative (n=67) control sera. Then, the ELISA was extended to sera (n=548) from HS aged 18-65 yrs old. Age-specific MCPyV-seroprevalence was investigated. Performance evaluation indicated that the assay showed 80% sensitivity, 91% specificity and 83.9% accuracy, with positive and negative predictive values of 94.3% and 71%, respectively. The ratio expected/obtained data agreement was 86%, with a Cohen’s kappa of 0.72. Receiver-operating characteristic (ROC) curves analysis indicated that the areas under the curves (AUCs) for the two peptides were 0.82 and 0.74, respectively. Intra-/inter-run variations were below 9%. The overall prevalence of serum IgGs anti-MCPyV in HS was 62.9% (345/548). Age-specific MCPyV-seroprevalence was 63.1% (82/130), 56.7% (68/120), 64.5% (91/141), and 66.2% (104/157) in HS aged 18-30, 31-40, 41-50 and 51-65 yrs old, respectively (p>0.05). Performance evaluation suggests that our indirect ELISA is reliable in detecting IgGs anti-MCPyV. Our immunological data indicate that MCPyV infection occurs asymptomatically, at a relatively high prevalence, in humans.

## Introduction

Merkel cell polyomavirus (MCPyV) is a small double-stranded oncogenic DNA virus belonging to the *Polyomaviridae* family (1, 2). This oncogenic polyomavirus is the causative infectious agent for Merkel Cell Carcinoma (MCC), which is a rare but aggressive skin tumor of neuroendocrine origin (3, 4). It has been reported that oncogenic MCPyV infection is associated with about 80% of MCCs (5). Integration of the MCPyV genome into human DNA is a key event for the MCC onset. Indeed, viral DNA integration can lead to mutations/chromosomal rearrangements. Another key mechanism is operated by two viral non-structural proteins with oncogenic potential, large T (LT) and small t (ST) antigens (5). Indeed, the expression of these viral oncoproteins modulate several cell signaling pathways, thereby leading to MCC, through a multistep process (6). Other MCPyV proteins include structural proteins named major capsid viral protein 1 (VP1) and minor capsid protein 2 (VP2) (6). Unlike other human polyomaviruses (PyVs) (1), MCPyV does not seem to express VP3 (7).

Previous studies have reported the presence of MCPyV DNA in blood and serum from healthy subjects (HS) (8–10). About 14% of peripheral blood mononuclear cells from pregnant females have been found to be MCPyV DNA-positive (8). Moreover, nearly 2% of sera from HS carry circulating MCPyV DNA (9). MCPyV DNA sequences have also been detected in cutaneous/nasal swabs, eyebrows, chorionic villi and adrenal glands (11–16). As it has been frequently detected in the skin from HS, MCPyV is considered a member of the human skin microbiota (17). Consistently, its transmission seems to occur *via* skin-to-skin contact leading to the virions being cutaneously spread (13). Despite MCPyV having high oncogenic potential characteristics, its infection seems to be widespread asymptomatically in different human populations. Soon after primary infection, MCPyV evokes a physiological immune response, then it is maintained in a latent/persistent state lifelong in immunocompetent hosts (18). Impairments of the host anti-viral immune response can promote MCPyV reactivation, which ultimately leads to the MCC onset, in rare cases (18). Immunocompromised organ transplant recipients and both oncologic and acquired immune deficiency syndrome (AIDS) affected patients, are more likely to develop MCC (19). Iatrogenic immune-impairment with biologics, such as monoclonal antibodies (abs), or with small molecules, such as Janus kinase (JAK) inhibitors employed in autoimmune diseases management (4, 20–22), i.e., rheumatic disorders, can also increase the risk of the MCC onset (4). When taken together, these data explain why this virus, widespread among humans, exerts its oncogenic potential only in a fraction of individuals who develop MCC.

Conflicting results have been reported on MCPyV seroprevalence in HS. Previous immunological studies have revealed widely differing prevalence rates, ranging from 55-87%, according to the study being considered (23–35). Immunological data concordantly indicate that primary MCPyV infection occurs early in life (23, 30), whilst about 40% and 60% of 1-4 and 5-10 year (yr) old children, respectively, test positive for circulating IgG abs against MCPyV (23, 24, 36, 37). Nearly all MCC patients carry circulating abs to MCPyV (30, 38, 39).

Current immunological assays for detecting serum IgGs against MCPyV comprise relatively laborious enzyme-linked immunosorbent assays (ELISAs). These assays mainly employ recombinant virus-like particles (VLPs) from MCPyV VP1, as antigens (40). More recent studies have reported the development of multiplex detection assays based on fluorescent bead technology, in combination with a glutathione S-transferase (GST) capture ELISA (41–43). Despite allowing abs against different viruses, including MCPyV, to be detected simultaneously, these systems employ viral capsomers as antigens for immunoreactions (41–43). Using VLPs could be considered a methodological limitation. Indeed, VLPs may increase the probability of cross-reactions between different viruses with a certain degree of homology, therefore hampering the result (26, 38, 44). In addition, VLP production is a difficult and laborious task, which requires MCPyV VP1 coding sequence cloning and protein *in vitro* synthesis using *Spodoptera frugiperda* insect cells, or recombinant bacteria. Furthermore, generated VLPs need to be purified and quantified. Only after following these time consuming steps, VLPs can be employed as antigens for immunoreactions (23, 26, 38). All considered, current VLPs-based immunological assays appear to be expensive, laborious and time consuming (23, 26). Given the current state of knowledge, it is important to have specific, rapid and low-cost methods, which allow abs against MCPyV to be identified unequivocally, in: (i) patients suffering from various diseases, including cancer, such as MCC; (ii) immunocompromised organ transplant recipients and AIDS patients; (iii) HS, who can be blood, stem cell and organ donors. Hence, there is progressive demand for more straight-forward and analytical assays to detect abs against MCPyV, especially in MCC-risk immunosuppressed individuals/patients.

The need for specific and rapid immunologic assays for MCPyV-seropositivity assessment prompted us to develop and validate a novel indirect ELISA to identify IgGs against MCPyV, without using recombinant proteins as antigens. To this purpose, two linear synthetic peptides, which mimic MCPyV VP1 and VP2, were designed using a computer-assisted approach to unequivocally recognize IgGs elicited against linear/conformational MCPyV VP1 and VP2 mimotopes/epitopes. Subsequently, an indirect ELISA was developed evaluating its performance in detecting serum anti-MCPyV IgGs, considering a number of criteria, such as sensitivity, specificity, efficiency, validity, repeatability/reproducibility and linearity, among other criteria, in a set of controls, i.e., MCPyV-positive and -negative sera. Receiver operating characteristic (ROC) curve analyses were also performed. The assay was then extended to sera from HS, ranging 18-65 yrs old. Exposure to MCPyV infection and age-specific MCPyV-seroprevalence in HS were therefore determined with our new indirect ELISA using VP mimotopes, as synthetic peptides.

## Materials and Methods

### Sera

Indirect ELISA diagnostic performance evaluation was performed on n=132 human control sera, which were previously analyzed for MCPyV-seropositivity using a VLPs-based ELISA test, as reported (26, 38). Controls comprised (i) MCPyV-negative [n=67, mean age ± standard deviation (SD), 53 ± (7) years] and (ii) MCPyV-positive healthy individuals [n=65, mean age ± SD, 50 ± (9) years]. Our assay was then validated on n=548 [mean age ± SD, 42 ± (13) years] sera from healthy subjects (HS) with unknown MCPyV serology taken from our serum collection (45–47). Anonymously collected sera were coded with age and gender indications. Written informed consent was obtained from subjects. The County Ethical Committee, Ferrara, Italy approved the project (ID:151078). Samples were stored at -80°C until testing.

### Computational Analyses

*In silico* analyses were performed to assess the reliability of the two linear synthetic MCPyV VP1 S and VP2 F peptides/mimotopes for detecting anti-MCPyV IgGs. Amino acid (a.a.) sequences of VP1 S (24 a.a.) and VP2 F (25 a.a.) peptides, are as follows:

VP1 S: NH_2_-NSPDLPTTSNWYTYTYDLQPKGSS-COOH;
VP2 F: NH_2_-SLSPTSRLQIQSNLVNLILNSRWVF-COOH.

Sequence analyses/alignments were carried out using the NCBI database and Clustal Omega tool (Hinxton, Cambridgeshire, UK). S and F peptides a.a. sequences were mapped on native MCPyV VPs to verify structural similarities. The monomeric form of MCPyV VP1 was obtained from the Protein data Bank (PDB, ID:4FMG) (48), while MCPyV VP2 was obtained *via* computational prediction carried out using the DNASTAR tool (Lasergene, Madison, WI). Molecular VPs visualizations were performed using the DNASTAR tool.

### Indirect ELISA

The indirect ELISA was developed and validated to detect specific IgGs against MCPyV. The initial concept of the assay has been reported for other PyVs (1, 47), while the herein newly employed assay was specifically conceived for MCPyV. S and F peptides were purchased from UFPeptides s.r.l., Ferrara, Italy. ELISA plates (Nunc-immuno plate, Thermo Fisher, Milan, Italy) were coated with 5 μg of selected peptide for each well, diluted in 100 μL of Coating Buffer 1X (Candor Bioscience, Wangen, Germany). Plates were left at 4°C for 16 h and then rinsed three times with Washing Buffer (Candor Bioscience, Wangen, Germany). Blocking phase was done using 200 μL/well of blocking solution (Candor Bioscience, Wangen, Germany) at 37°C for 90 min (47). Wells were rinsed three times. Each well was covered with 100 μL containing 1:20 diluted sera in low cross-buffer (Candor Bioscience, Wangen, Germany). Control sera in each plate were: (i) positive controls, three immune sera derived from patients with MCPyV-positive MCC; (ii) negative controls, three MCPyV-negative sera. Subsequently, plates were rinsed three times, before adding secondary Ab. A goat anti-human IgG heavy (H) and light (L) chain specific peroxidase conjugate (Calbiochem-Merck, Darmstadt, Germany) was diluted 1:10,000 in Low Cross-Buffer. The reaction mixture was incubated at room temperature (RT) for 90 min (47). Wells were rinsed three times, and then 100 μL of 2,2′-azino-bis-3-ethylbenzthiazoline-6-sulfonic acid (ABTS) solution (Sigma-Aldrich, Milan, Italy) was added to each well. After 45 min at RT plates were read by spectrophotometer (Thermo Electron Corp., model Multiskan EX, Vantaa, Finland) at a wavelength (λ) of 405 nm. Color intensity in wells was determined by optical density (OD) reading. The cutoff of each peptide was determined in each ELISA run, as the mean of the OD readings of n=3 negative control sera, adding three standard deviations of mean (mean +3 SDs) (49, 50), as widely described previously for other ELISAs (47, 51, 52). Sera were considered MCPyV-positive when reacting to both S and F synthetic peptides, in three replica experiments carried out by independent operators.

### Indirect ELISA Performance Evaluation

Indirect ELISA performance in detecting serum anti-MCPyV IgGs was evaluated on MCPyV-negative (n=67) and MCPyV-positive (n=65) control sera. The following sample conditions were considered: true positive (TP, the number of positive samples according to previous analyses), false positive (FP, the number of positive samples with our assay and negative with previous analyses), true negative (TN, the number of negative samples according to previous analyses) and false negative (FN, the number of negative samples with our assay and positive with previous analyses) (53). Assay performance was then assessed computing a number of set-up criteria (53–58), as follows: sensitivity [Se, TP/(TP+FN)], specificity [Sp, TN/(TN+FP)], positive predictive value [PPV, TP/(TP+FP)], negative predictive value [NPV, TN/(TN+FN)], validity [as (Se+Sp)/2], accuracy [Se*Prevalence+Sp*(1−Prevalence) and the overall efficiency (Ef)] [or relative agreement, as (TP+TN)/(TP+TN+FP+FN) (**Table 1**) (53). Assay accuracy was evaluated considering the following combined measures: Youden’s Index (J, Se+Sp-1) (54), positive likelihood ratio [LR+, as Se/(1-Sp)], negative likelihood ratio [LR-, (1-Se)/Sp] (52, 53). Furthermore, Cohen’s kappa coefficient (κ) was used to evaluate the agreement between expected and obtained results (56). The agreement was interpreted as poor (κ≤0), slight (0<κ≤0.20), fair (0.21<κ≤0.40), moderate (0.41<κ≤0.60), substantial (0.61<κ≤0.80), and near-perfect (0.81<κ≤1.0) (56). The performance of each S and F peptide was also analyzed by building receiver-operating characteristic (ROC) curves (59). ROC curves were used to calculate the area under the curve (AUC) for each peptide by comparing expected/obtained results (57). The AUC provides a global summary of statistic test robustness by considering the assay as: non-informative (AUC=0.5), low (0.5<AUC ≤ 0.7), moderate (0.7<AUC ≤ 0.9) and high (0.9<AUC<1) accurate and perfect (AUC=1) (58). Concordance in ODs between S and F peptides was evaluated on MCPyV-negative/-positive control sera using Spearman correlation r coefficient (with the 95% CI) (60).

**Table 1:** Indirect ELISA diagnostic performance criteria.

Criteria	Values
Sensitivity (Se)	80.00%
Specificity (Sp)	91.05%
Positive Predictive Value (PPV)	94.32%
Negative Predictive Value (NPV)	71.03%
Efficiency (Ef)	85.61%
Validity	85.52%
Accuracy	83.87%
Youden’s Index (J)	0.71
Likelihood ratio (LR+)	8.93%
Likelihood ratio (LR-)	0.22
Agreement	85.60%
Cohen’s Kappa value (κ)	0.72

Indirect ELISA diagnostic performance criteria obtained by testing MCPyV-negative (n=67) and -positive (n=65) control sera.

### Determining Indirect ELISA Precision and Dilutional Linearity

Indirect ELISA precision was assessed by evaluating assay repeatability (intra-assay variability) and reproducibility (inter-assay variability). Intra-assay repeatability was evaluated by measuring the coefficient of variations (CVs) for 90 repeats of 30 sera for each MCPyV VP1 S and VP2 F peptide. Sera with high (n=10, OD>0.17 for S peptide and OD>0.4 for peptide F), medium (n=10, 0.12<OD<0.17 for S peptide and 0.2<OD<0.4 for F peptide) and low (n=10, OD<0.12 for S peptide and OD<0.2 for F peptide) ODs were selected. Inter-assay reproducibility was evaluated by performing 3 independent runs for each peptide, by different operators. Adequate rates were determined for intra-assay CV to 10% and inter-assay CV to 15% (61). In order to determine assay dilutional linearity (accuracy), 3 samples with high ODs (obtained with the assay used herein) and diluted 1:20, 1:40 1:80, 1:160, 1:320, 1:640, 1:1,280 and 1:2,560 were selected and tested in triplicate.

### Statistical Analysis

A two-sided chi-square test was used to statistically analyze MCPyV seroprevalences (46, 62). Values were analyzed using the D’Agostino Pearson normality test and then parametric and non-parametric tests were applied according to normal and non-normal variables, respectively (60, 63). Linear regression of correlation coefficient (R^2^) (with the 95% CI) was computed to evaluate assay linearity (60). Data were analyzed using the one-way Anova analysis and Kruskal-Wallis multiple comparison test (OD mean, 95%CI). Statistical analyses were carried out using MedCalc Statistical Software version 16.2.1 (MedCalc Software bvba, Ostend, Belgium; https://www.medcalc.org) and Graph Pad Prism version 4.0 for Windows (Graph Pad, La Jolla, USA) (64). P values <.05 were considered statistically significant (65).

## Results

### Computer Assisted Analyses

**Figure 1:**
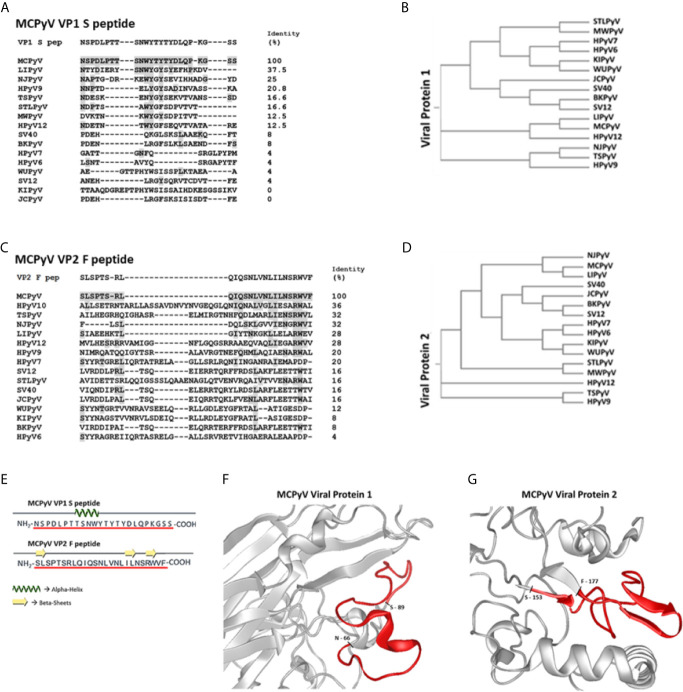
Amino acid sequences of S/F peptides, polyomaviruses phylogenetic trees and partial VP1/2 structures visualizations. Panels **(A, C)** show identity between Merkel cell polyomavirus (MCPyV)-specific VP1 S and VP2 F peptides and VP1 and VP2 from human/simian polyomaviruses (PyVs). Both S and F peptides were selected on the basis of their low homology with corresponding a.a. sequences from VP1 and VP2 from known human/simian PyVs. VP1 and VP2 a.a. sequences/IDs were obtained from (https://www.ncbi.nlm.nih.gov/protein/) and aligned with Clustal Omega software. Panels **(B, D)** show PyVs phylogenetic trees according to VP1 and VP2 from human/simian PyVs comparisons. Panel **(E)**: computational analyses indicate that both peptides show several random coiled domains with 1 alpha helix (S peptide) and 3 beta-sheets (F peptide). Panels **(F, G)**: partial view of S and F peptides (red) within the three-dimensional (3D) models of VP1 and VP2. The partial VP2 representation is based on a structural prediction obtained using DNASTAR software.

Two linear synthetic peptides, named MCPyV VP1 S and VP2 F (S and F peptides), which mimic the immunoreactive MCPyV VP1 and VP2 epitopes, respectively, were designed and employed in indirect ELISA as mimotopes to detect anti-MCPyV IgGs. The identity between a.a. strains from S and F peptides and corresponding VP native polyomaviruses (PyVs) from known 16 human/simian PyVs, including MCPyV and its different strains, was assessed (**Figures 1A–D**, **Supplementary Material 1**). The S and F peptides a.a. sequences were 100% concordant with MCPyV VP1 and VP2 strains, respectively (**Figures 1A, C**). The S peptide a.a. sequence was 100% identical to the corresponding VPs from the five main MCPyV isolates, including North American (MCC350 or EU375803.1 and MCC339, or EU375804.1), Japanese (TKS and FJ464337), European (MKL-1 or FJ173815), and Chinese (HB039C or KC571692.1) isolates (4, 66). Likewise, the F peptide a.a. chain was 100% identical to that of VPs from North American and European isolates (4, 66). Moreover, the a.a. sequence of F peptide was 96% (24/25 a.a.) concordant with that of VPs from the Japanese and Chinese isolates (F to S at the last a.a. position of the peptide, n.25) (4, 66), as well as with that from AHW79949 and AWG42110 VP strains (T to I at a.a. position n.5 of the peptide in both cases) (67, 68).The identity with the VP native strains from other 15 PyVs was below 37.5% and 36% for S and F peptide, respectively. In addition, a total of 12 and 8 different PyV types shared less than 16.6% and 16% of identity with the a.a. strains from S and F peptides, respectively (**Figures 1A, C**). Both S and F peptides also differ from VP native strains from 15 PyVs, due to the frequent presence (or absence) of additional a.a. residues within their sequence (**Figures 1A, C**).

Computational analyses indicate that S peptide corresponds to a.a. 66-89 of MCPyV VP1, while F peptide lies within a.a. 153-177 of MCPyV VP2. In addition, S peptide also encompasses the VP1 BC surface loop (69). Notably, a.a. sequences from S and F peptides were characterized toward a stable secondary structure formation. Indeed, S peptide forms a stable secondary structure from a.a. 8 to a.a. 11, i.e., _8_TSNW_11_, where an alpha helix domain is found, surrounded by two random coil secondary structures (**Figure 1E**). F peptide has 3 random coil secondary structures, while it presents three short beta sheet domains from a.a. 1 to a.a. 2, i.e., _1_SL_2_, from a.a. 18 to a.a. 19, i.e., _18_IL_19_, and from a.a. 22 to a.a. 23, i.e., _22_RW_23_ (**Figure 1E**). Tertiary structures of MCPyV VPs were retrieved from the available PDB structures (VP1) and computationally predicted by using the DNASTAR tool (VP2). The three-dimensional graphic rendering of S and F peptides mapped onto the inferred MCPyV VP1 and VP2 native structures, respectively, indicated that these regions are exposed on VP surfaces (**Figures 1F, G**). In addition, S peptide is also located within the VP1 BC surface loop (69). The spatial configuration of S and F peptides may thus represent natural short docking sites for serum IgGs against both linear and conformational antigens.

### Indirect ELISA Performance Parameters Evaluation

**Figure 2:**
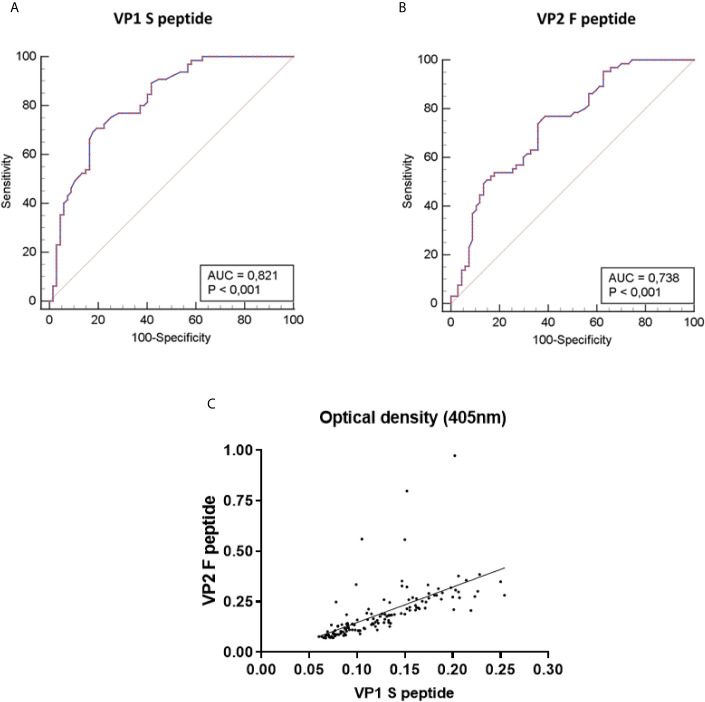
Receiver-operator characteristic (ROC) curves and correlation of Optical density (OD) values of the indirect ELISA. ROC curves were built based on optical density (OD) values obtained on MCPyV-negative (n=67) and -positive (n=65) control sera, for both MCPyV VP1 S and VP2 F peptides. The values for the area under the ROC curve (AUC) were 0.821 for VP1 S peptide **(A)** and 0.738 for VP2 F peptide **(B)**. The diagonal line shows an AUC value of 0.5 which is representative of a worthless test. The difference between AUCs for both S and F peptides resulted statistically significantly different from that of a worthless test (AUC=0.5, P<0.001). **(C)** The concordance in ODs between the S and F peptides was evaluated on the entire set of MCPyV-negative/-positive control sera (n=132) using Spearman correlation analysis. Concordance between VP1 S peptide and VP2 F peptide was good, with an r of 0.8723 and a p<0.0001.

Developing our indirect ELISA provided for an evaluation of its performance in detecting serum anti-MCPyV IgGs on MCPyV-negative (n=67) and -positive (n=65) 1:20 diluted control sera. The evaluated criteria, computed by comparing expected and obtained results, are depicted in **Table 1**. The assay demonstrated 80.00% (95% CI: 68.23–88.89%) sensitivity and 91.05% (95% CI: 81.52–96.64%) specificity, with a PPV of 94.32% (95% CI: 88.45-97.29) alongside a NPV of 71.03% (95% CI: 59.97-80.03%), in detecting serum anti-MCPyV IgGs (**Table 1**). Likewise, the efficiency, validity and accuracy criteria resulted as 85.61%, 85.52% and 83.87% (95% CI: 76.46-89.68%), respectively (**Table 1**). The evaluation also indicated that J, LR+ and LR- were 0.71, 8.93 (95% CI: 4.12-19.35) and 0.22 (95% CI: 0.13-0.35), respectively (**Table 1**). Agreement between expected and obtained results resulted as 85.60%, with a κ of 0.72 (95% CI: 0.61-0.84) (**Table 1**). ROC curves were built based on ODs obtained on MCPyV-positive and -negative control sera, for both S and F peptides. AUC resulted as 0.821 (95% CI: 0.745 to 0.882) for the peptide S and 0.738 (95% CI: 0.654 to 0.811) for the peptide F (**Figure 2**). The difference between AUCs for both S and F peptides was statistically significant compared to that of a worthless test (AUC=0.5, P<0.001) (**Figure 2**). ODs obtained with S and F peptides on same control sera were also compared. A good correlation between ODs for S and F peptides was found to be, using a Spearman correlation coefficient r, 0.8723 (p<0.0001) (**Figure 2C**).

### Indirect ELISA Repeatability, Reproducibility, and Dilutional Linearity Evaluation

To assess indirect ELISA precision in terms of repeatability (intra-assay variability) and reproducibility (inter-assay variability), CVs were measured on n=30 MCPyV-positive sera which were tested in triplicate in one run and in three independent experiments, for each S and F peptide (**Table 2**). Sera were selected and stratified according to the ODs obtained with both peptides in sera with low (n=10), medium (n=10) and high (n=10) ODs. A total of 180 repeats were done. Intra-assay repeatability CVs for low, medium and high OD groups were 5.03%, 7.28% and 7.45% for S peptide, respectively, and 6.41%, 6.02% and 7.17% for F peptide, respectively (**Table 2**). Inter-assay reproducibility CVs were 4.40% and 5.65% for the low OD group, 7.09% and 8.05% for the medium OD group and 7.86% and 8.85% for the high OD group, for S and F peptide, respectively (**Table 2**).

**Table 2:** Indirect ELISA intra-assay and inter-assay coefficient of variations (CVs).

Peptides	Groups		
	Low	Medium	High
	(n=10)	(n=10)	(n=10)
**S peptide**			
* Intra-assay (n=3 replicates)*			
Mean	0.062	0.156	0.178
SD	0.006	0.051	0.057
CV (%)	5.03	7.28	7.45
* Inter-assay (n=3 replicates)*			
Mean	0.063	0.161	0.186
SD	0.005	0.049	0.06
CV (%)	4.4	7.09	7.86
**F peptide**			
* Intra-assay (n=3 replicates)*			
Mean	0.077	0.259	0.57
SD	0.021	0.087	0.21
CV (%)	6.41	6.02	7.17
* Inter-assay (n=3 replicates)*			
Mean	0.078	0.267	0.592
SD	0.022	0.092	0.216
CV (%)	5.65	8.05	8.85

Indirect ELISA intra-assay and inter-assay coefficient of variations (CVs) evaluated on three set of control sera with high (n=10), medium (n=10) and low (n=10) optical density levels, for both Merkel cell polyomavirus VP1 S and VP2 F peptides. SD, standard deviation; CV, coefficient of variation.

**Figure 3:**
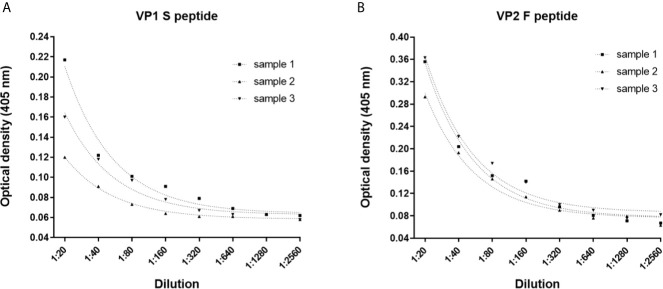
Dilutional linearity of the indirect ELISA. Optical Density (OD) response to serial dilutions (1:20, 1:80, 1:160, 1:320, 1:640, 1:1,280 and 1:2,560) of n=3 MCPyV-positive sera presenting known high ODs. Each dilution was assayed in triplicate for each MCPyV VP1 S and VP2 F peptide, and ODs and sera dilutions were compared by linear regression analysis. **(A)** Good correlation between ODs and dilutions was found for VP1 S peptide with an R^2^ of 0.9781 (p<0.0001), 0.9925 (p<0.0001) and 0.9809 (p<0.0001) for samples #1, #2 and #3, respectively. **(B)** Good correlation between ODs and dilutions was found for VP2 F peptide, with an R^2^ of 0.9793 (p<0.0001) for sample #1, 0.9851 (p<0.0001) for samples #2 and 0.9853 (p<0.0001) for samples #3.

The dilutional linearity (accuracy) of the indirect ELISA was determined by performing serial dilutions, from 1:20 to 1:2,560, using n=3 MCPyV-seropositive samples with known high ODs when tested using both S and F peptides. For each peptide, dilutions were assayed in triplicate, and ODs and dilution values were compared by linear regression analysis. The assay had a high correlation between ODs and sample dilutions when the S peptide was employed, with an R^2^ of 0.9781 (p<0.0001), 0.9925 (p<0.0001) and 0.9809 (p<0.0001) for samples #1, #2 and #3, respectively (**Figure 3A**). The assay also showed a high correlation between ODs and sample dilutions when using F peptide, with an R^2^ of 0.9793 (p<0.0001) for sample #1, 0.9851 (p<0.0001) for samples #2 and 0.9853 (p<0.0001) for samples #3 (**Figure 3B**).

### Detection of Serum IgG Antibodies Against MCPyV by Indirect ELISA in HS

The novel indirect ELISA employing S and F peptides/mimotopes was developed to assess whether serum samples from HS (n=548) contain IgG Abs which react to MCPyV antigens and to determine the distribution of MCPyV infection in HS. Sera from HS which react to S and F peptides reached an overall similar prevalence, of 72.4% (397/548) and 67.8% (372/548), respectively (**Table 3**, p>0.05). Conversely, negative sera for the S peptide failed to react with F peptide. Few serum samples were exceptions. Indeed, 4.9% (27/548) of sera were negative for S peptide, while testing positive for F peptide. Similarly, 9.5% (52/548) of sera resulted negative for F, while testing positive for S peptide. Combining MCPyV-positive sera, both for S and F peptides, overall prevalence in HS was 62.9% (345/548). Combined S and F peptides reactivity was then determined in age-stratified HS, i.e., 18-30 yrs, 31-40 yrs, 41-50 yrs, and 51-65 yrs, and rates were compared. A prevalence pattern of combined S and F corresponding to 63.1% (82/130), 56.7% (68/120), 64.5% (91/141), and 66.2% (104/157), was found in cohorts of HS aged 18-30, 31-40, 41-50 and 51-65 yrs old, respectively (**Table 3**, p>0.05).

**Table 3:** IgG antibodies seroprevalence against MCPyV VP1 S and VP2 F peptides in heathy subjects (HS).

Age yrs	Number of samples	Number of positive samples (%)			
		Male	VP1 S	VP2 F	VP1 S+VP2 F
		(%)			
18-30	130	20.8	92 (70.8)	90 (69.2)	82 (63.1)
31-40	120	36.7	79 (65.8)	72 (60)	68 (56.7)
41-50	141	39	104 (73.8)	102 (72.3)	91 (64.5)
51-65	157	53.5	122 (77.7)	108 (68.8)	104 (66.2)
Total	548	38.3	397 (72.4)	372 (67.8)	345 (62.9)

Serum samples (n=548) were from healthy subjects (HS). Statistical analyses were performed using the two-sided chi-square test. No statistical differences were detected among age-stratified groups (p>0.05).

To assess any association between MCPyV infection and gender, the presence of Abs against MCPyV were determined in males (n=210) and females (n=338) HS, then rates were compared. It turned out that there was a similar prevalence of anti-MCPyV Abs in both males and females which resulted as 65.2% (137/210) and 61.5% (208/338) (p>0.05), respectively.

**Figure 4:**
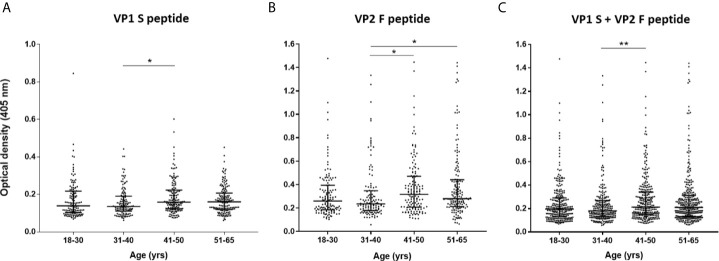
Serologic profiles of serum antibody reactivity to MCPyV peptides in age-stratified healthy subjects (HS). Analyses were performed with VP1 S **(A)**, VP2 F **(B)** peptides and for combined VP1 S and VP2 F **(C)** in healthy subjects (HS). Immunologic data are from age-stratified HS (n=548) and results are presented as optical density (OD) value readings at λ 405 nm for serum samples assayed in indirect ELISA. In the scatter dot plot, each dot represents the dispersion of ODs for each sample. The median is indicated by the line inside the scatter plot with the interquartile range (IQR) in age-stratified HS, i.e., 18-30 yrs (n=130), 31-40 yrs (n=120), 41-50 yrs (n=141), and 51-65 yrs (n=157). (A) *p<0.05; (B) *p<0.05 for 41-50 *vs* 31-40 and for 51-65 *vs* 31-40; (C) **p<0.01.

**Figure 5:**
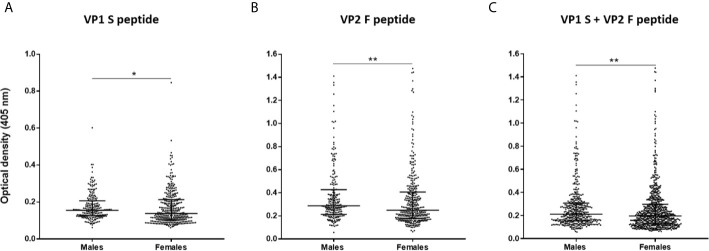
Serologic profiles of serum antibody reactivity to MCPyV peptides in gender-stratified healthy subjects (HS). Analyses were performed with VP1 S **(A)**, VP2 F **(B)** peptides and for combined VP1 S and VP2 F **(C)** in gender-stratified HS. Immunologic data derived from males (n=210) and females (n=338) HS. Results are presented as OD value readings at λ 405 nm for serum samples assayed in indirect ELISA. In the scatter dot plot, each dot represents the dispersion of ODs for each sample. The median is indicated by the line inside the scatter plot with the interquartile range (IQR) for each group of subjects analyzed. (A) *p<0.05; (B) **p<0.01; (C) **p<0.01.

Serological profiles of serum IgG Abs reactivity to S and F peptides alone and to combined S and F peptides are shown in **Figures 4** and **5**. Immunological data are from the entire cohort of HS (n=548) whereas results are presented as OD readings at λ 405 nm. The dispersion of ODs is reported in the scatter dot plot, where each dot represents one OD for each serum sample tested (**Figures 4** and **5**). Median [interquartile range (IQR)] ODs were then determined in age-stratified HS, i.e., 18-30 yrs, 31-40 yrs, 41-50 yrs, and 51-65 yrs and compared. The ODs for S peptide resulted as higher in HS aged 41-50 yrs (0.15 OD, 0.12-0.22) than in those aged 31-40 yrs (0.13 OD, 0.11-0.19, p<0.05) (**Figure 4A**). The ODs for F peptide were higher in sera from both HS aged 51-65 yrs (0.27 OD, 0.21-0.44) and 41-50 yrs (0.31 OD, 0.20-0.47) than in those from 31-40 yrs (0.23 OD, 0.17-0.34) (p<0.05) (**Figure 4B**). Furthermore, higher ODs for combined S and F peptides were detected in 41-50 yrs group (0.21 OD, 0.15-0.34) than in 31-40 yrs group (0.18 OD, 0.13-0.26, p<0.01) (**Figure 4C**). Serum Ab reactivity to S and F peptides was also evaluated in gender-stratified HS (**Figure 5**). Median (IQR) OD values were therefore determined in males (n=210) and females (n=338) and compared. The ODs for S peptide in males (0.15 OD, 0.12-0.2) resulted higher compared to those in the female group (0.13 OD, 0.1-0.21, p<0.05) (**Figure 5A**). Similarly, ODs for F peptide were higher in sera from males (0.28 OD, 0.21-0.42) than in those from females (0.24 OD, 0.17-0.40, p< 0.01) (**Figure 5B**). Consistently, higher ODs for combined S and F peptides were detected in males (0.21 OD, 0.15-0.3) than in females (0.19 OD, 0.12-0.29, p<0.01) (**Figure 5C**).

## Discussion

In this study, a reliable indirect ELISA based on synthetic peptides used as MCPyV VP mimotopes was developed and validated to detect IgGs which react to MCPyV VP antigens. The assay was set up using MCPyV-negative/-positive control sera, while its validation was performed on sera from HS aged 18-65 yrs of age with unknown MCPyV-serology. HS age-specific MCPyV-seroprevalence was then determined. The need for more specific and rapid MCPyV-focused immunoassays prompted us to set-up and validate this new method.

*In silico* analyses were performed to assess the theoretical reliability of MCPyV VP1 S and VP2 F peptides as mimotopes/antigens. The S and F a.a. chains (i) are located within MCPyV VP1 and VP2 proteins, respectively, (ii) show shapes which are comparable to those of the native VPs, (iii) are exposed to the environment. In addition, S peptide is located on the VP1 BC surface loop (69). Both S/F positions may thus represent natural antigens establishing sites for immunoreactions. These computational data suggest that these peptides may be used for detecting IgGs to MCPyV by indirect ELISA, as they meet the requirement of mimotopes (47).

Indirect ELISA performance in detecting serum anti-MCPyV IgGs was then assessed on MCPyV-negative/-positive control sera. Controls were tested to compute a number of criteria which are commonly used in determining assay performances, including ELISA (53–58). Our assay proved to have adequate sensitivity (80%) and specificity (91%), with good PPV (94%) and NPV (71%), in detecting anti-MCPyV Abs, as described (53, 70, 71). Our method was also efficient, valid and accurate, as indicated by suitable rates ranging 84-86%, for the corresponding criteria (53). The expected/obtained results agreement was 86%, with a κ of 0.72, which is within the 0.61-0.80 range of substantial agreement (56, 57, 72). Acceptable J (0.71), LR+ (8.93) and LR- (0.22) values were also determined (54, 55, 73). In particular, a J towards 1 excludes the existence of false positives/negatives (54, 73), while the higher (or lower) LR of a positive (or negative) test is, the more reliable is the result of the assay undergoing development (55). These trustworthy values cumulatively indicate a relevant correlation between expected and obtained results (70), thus underlining that our assay achieves suitable prerequisites for anti-viral IgGs detecting purposes (53, 56, 70, 71). The performance of each S and F peptide was assessed using ROC curve analysis, which is a key evaluating tool (59) and corresponding AUCs were calculated (57). AUCs of S (0.82) and F (0.74) peptides were both within the adequate 0.7-0.9 range (55, 57), and higher than that of a worthless test (0.5, p<0.001) (74). Spearman correlation analysis revealed that S and F peptide ODs were highly correlated when compared, with an r of 0.87 (p<0.0001). These data indicate that our assay is consistent in distinguishing positive and negative sera, and that both peptides can be exploited simultaneously. To evaluate assay precision in terms of repeatability and reproducibility, intra-/inter-assay CVs were measured (60). Marginal variations were found, as the variance between replicates reached the means over replicates and CVs were lower than 9%, thus below the acceptable 10-15% range (57, 61, 74). The assay, described herein, provides for the repeatable and reproducible detection of anti-MCPyV Abs and may be used for patient longitudinal monitoring, as abs could be recorded over time (75). For assay dilutional linearity, a remarkable correlation between ODs and sera dilutions was found, as linear regression analyses revealed an R^2^ ranging 0.98-0.99 (p<0.0001). This evidence indicates that our method is also accurate (76, 77). These performance criteria cumulatively demonstrate that our indirect ELISA is reliable, sensitive and specific in detecting IgG Abs to MCPyV.

Following set-up steps, we extended our assay on sera from HS aged 18-65 yrs old, with unknown MCPyV serology, while samples were studied for reactivity to MCPyV antigens. Indeed, the main objective of this study was to assess whether sera from HS carry IgGs against MCPyV. Sera were considered positive when reacting to both S and F peptides (47).

The overall seroprevalence of MCPyV in the entire cohort of HS was 62.9%. This finding indicates that MCPyV circulates widely in the human population, while its infection is asymptomatic. MCPyV seroepidemiology in the healthy population is highly variable across different studies, ranging 46-87% (23–35). Notably, our results are in agreement with previous works reporting a prevalence ranging from 55% to 68%, in healthy individuals with a comparable age range to that of our study population (29, 35, 78, 79). However, other reports have described relatively high rates in adults, within 77-87% (23, 26, 36, 80). Although the immunoassay described herein does not allow our data and previous data to be adequately compared, variations in prevalence may reflect methodological disparities among assays (23, 26). Previous high MCPyV serological rates have been mainly obtained with ELISAs based on VLPs as antigens. As different PyVs show a certain degree of homology (26, 38), data-invalidating cross-reactions cannot be excluded, when using VLPs. The S peptide a.a. chain is 100% concordant with that from VP native strains belonging to the five main MCPyV isolates, including North American, Japanese, European and Chinese isolates (4, 66). The F peptide a.a. sequence is 100% identical with that to that of VPs from the North American and European isolates (4, 66), while it presents 96% of similarity when compared with VPs from the Japanese and Chinese isolates and with AHW79949 and AWG42110 VP strains (4, 66–68). Notably, sequence homologies for VPs from other PyVs were found to be remarkably low. This evidence supports the view that our assay is reliable in detecting circulating IgGs against MCPyV.

Analyses on age-stratified HS indicate that, although no statistical differences were found, MCPyV seroprevalence was slightly higher in the two older (41-50/51-65 yrs) groups, reaching 64.5 and 66.2%, respectively. MCPyV seroprevalence in healthy individuals has previously been found to increase with age (9, 24, 29–32, 34, 36, 79), while no age-related variations have also been reported (23, 26, 79, 80). A potential age-dependent seroprevalence pattern may imply that, despite the early seroconversion (35, 43), MCPyV infection most likely occurs throughout life (29, 34). Moreover, as MCPyV establishes a persistent infection, this kind of infection may also provide a permanent source of immune antigens resulting in a continuous/lifetime production of anti-viral IgGs (79). Notably, despite all HS having high ODs, the two older groups showed the highest levels (p<0.05). These findings, in agreement with those previously reported (23, 32, 34), may indicate that HS carry high levels of anti-MCPyV IgGs, which increase with age. IgGs may rise in an age-dependent manner as a result of the viral reactivation or reinfection with different strains and the following gradual production of antigenic MCPyV VPs, which is a frequent viral-related process in adulthood (81). Progressive viral reactivation during life may lead to viremia/immunoresponse (82), as MCPyV proteins are gradually exposed to the immune system.

Although a slightly higher number of sera from females was tested herein, gender-stratified HS proved no differences in abs reactivity to MCPyV between males and females (p>0.05). This finding may suggest that MCPyV infection is equally distributed without disparities based on gender, as theorized (23, 26, 34–36, 79, 80). By contrast, in this study, males showed higher ODs than females. This may imply that, although similar in term of prevalence, differences in exposure, or susceptibility to MCPyV infection may potentially exist in the HS studied herein. Higher anti-MCPyV IgG levels and/or seroprevalence rates have been described in healthy males compared to females (29, 83). Further studies are needed to clarify this issue.

Although the role of MCPyV in MCC is well-known (28), current MCPyV immunoassays are far from the clinical routine. These methods can necessitate several difficult tasks, as they require VLPs as antigens. This feature provides challenging/prolonged steps before ELISA protocol execution *per sé* (23, 26, 38, 40). On these grounds, our new assay, using synthetic peptides, which is specific, rapid, and reliable in detecting Abs to MCPyV, may be used in routine clinical laboratory analysis. MCC-risk individuals/patients including blood donors/recipients, immunocompromised organ transplant recipients and oncologic/AIDS patients (19), as well as patients under iatrogenic immune compromization with biologics or with JAK inhibitors (4), can benefit from our assay. Indeed, monitoring of Abs against MCPyV is crucial for ensuring a good outcome for patients, by preventing MCC onset. In addition, since MCPyV LT/ST play an important role in MCC carcinogens, evaluating the presence of IgGs against these oncoproteins in the aforementioned classes of individuals/patients might present clinical relevance. Our immunoassay can potentially be extended by employing novel synthetic linear peptides/mimotopes mimicking MCPyV LT/ST antigens, as previously performed for other PyVs (1). The simultaneous evaluation of IgGs against both MCPyV VPs and LT/ST antigens will allow a more comprehensive understanding of the impact of oncogenic MCPyV infection in the healthy population, in immunosuppressed patients and in MCC at risk patients. These experiments are feasible, and they could be part of next investigations.

In conclusion, developing methods to determine MCPyV-seropositivity is urgently needed in order to assess the impact of MCPyV infection in humans. In this study, a reliable indirect ELISA for the detection of serum anti-MCPyV IgGs in HS was successfully set-up and validated. *In-silico* analyses indicated that linear peptides may specifically recognize IgGs generated against linear/conformational MCPyV VPs. Assay performance criteria, including sensitivity, accuracy, specificity, reliability, as well as other criteria, were thoroughly evaluated in MCPyV-positive/-negative controls and resulted as adequate. Assay reliability was demonstrated by investigating the presence of IgGs to MCPyV in HS aged 18-65 yrs old. Abs to MCPyV resulted as detectable in sera from HS, without age-related variations. We may infer that MCPyV is circulating asymptomatically at a relatively high prevalence in humans.

## Data Availability Statement

The raw data supporting the conclusions of this article will be made available by the authors, without undue reservation.

## Ethics Statement

The studies involving human participants were reviewed and approved by The County Ethical Committee, Ferrara, Italy approved the project (ID:151078). The patients/participants provided their written informed consent to participate in this study.

## Author Contributions

Conceptualization, MT and JR. Methodology, CM and JR. Software, CM, JR, and LO-G. Validation, CL, ET, PG. formal analysis, CM, ET, LO-G. investigation, CM and MI. resources, ES and MG. Data curation and statistical analysis, CM. Writing-original draft preparation, CM and JR. Writing—review and editing, MT, FM, MG, and AT. Visualization, CM, CL, and EM. Supervision, JR and MT. Funding acquisition, JR and MT. All authors contributed to the article and approved the submitted version.

## Funding

This work was supported, in part, by grant IG 21956 (to JR) and by grant IG 21617 (to MT) from the Associazione Italiana per la Ricerca sul Cancro (AIRC), Milan, Italy.

## Conflict of Interest

The following authors CM, CL, ET, EM, FM, MT and JR are holders of the the Italian patent application number 102020000021235 (I0188839) BRE-mma, filed on September 8, 2020. Data of this work were enclosed, in part, in the aforementioned Italian patent.

The remaining authors declare that the research was conducted in the absence of any commercial or financial relationships that could be construed as a potential conflict of interest.
