# Supplementary material for: Serum Antibodies Against the Oncogenic Merkel Cell Polyomavirus Detected by an Innovative Immunological Assay With Mimotopes in Healthy Subjects
Source: Front Immunol. 2021 Jun 8;12:676627. doi: 10.3389/fimmu.2021.676627 (PMC8217635; doi:10.3389/fimmu.2021.676627)
Supplement: Supplementary file 1 [file DataSheet_1.docx]

Supplementary Material

# Supplementary Data

**GenBank Identity Numbers (IDs) of the Viral proteins 1/2/3 (VP1 and VP2/3) belonging to the known sixteen human/simian polyomaviruses (PyVs).**

1. JC polyomavirus (JCPyV, VP1 GenBank Identity Number (ID): QBX89124.1, VP2 ID: AFH57192.1).
2. BK polyomavirus (BKPyV, VP1 ID: AHU87368.1, VP2 ID: AUF70041.1).
3. Simian Virus 40 (SV40, VP1 ID: ABU62649.1, VP2 ID: ABU62647.1).
4. Trichodysplasia spinulosa-associated polyomavirus (TSPyV, VP1 ID: AID54934.1, VP2 ID: AID54932.1).
5. Simian Virus 12 (SV12, VP1 ID: ABD92876.1, VP2 ID: ABD92874.1).
6. KI polyomavirus (KIPyV, VP1 ID: ACR43495.1, VP2 ID: QCQ73648.1).
7. WU polyomavirus (WUPyV, VP1 ID: AVK92965.1, VP2 ID: ACD76100.1).
8. STL polyomavirus (STLPyV, VP1 ID: YP_007354884.1, VP2 ID: YP_007354882.1).
9. MW polyomavirus (MWPyV, VP1 ID: AFN43006.1, VP2 ID: AFN43004.1).
10. Human polyomavirus 7 (HPyV7, VP1 ID: AXF48237.1, VP2 ID: AXF48235.1).
11. Human polyomavirus 6 (HPyV6, VP1 ID: AMQ36137.1, VP2 ID: AMQ36135.1).
12. Human polyomavirus 9 (HPyV9, VP1 ID: QCB66181.1, VP2 ID: QCB66179.1).
13. Human polyomavirus 12 (HPyV12, VP1 ID: YP_007684355.2, VP2 ID: AGH58113.1).
14. New Jersey polyomavirus (NJPyV, VP1 ID: YP_009030020.1, VP2 ID: YP_009030018.1).
15. Merkel cell polyomavirus (VP1 ID: AFC36093.1, VP2 ID: AFC36094.1).
16. Lyon IARC polyomavirus (LIPyV, VP1 ID: YP_009352870.1, VP2 ID: YP_009352868.1).

**
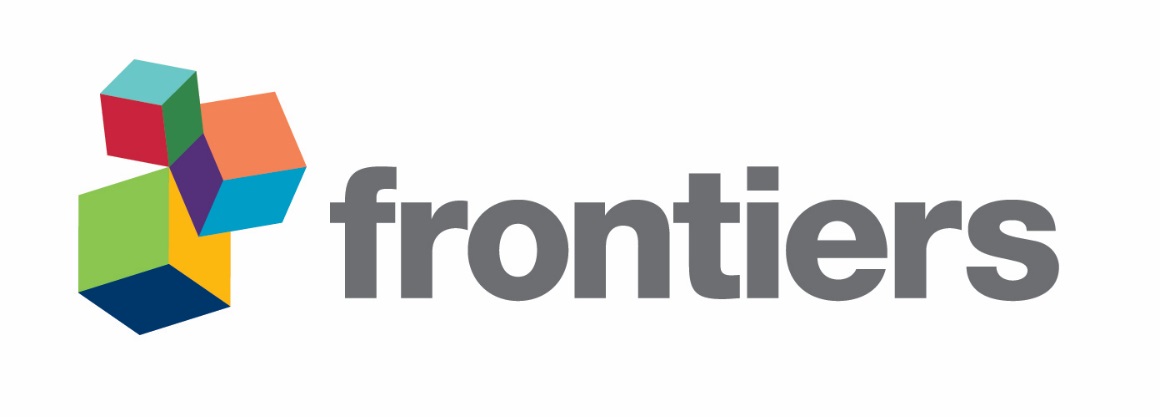
**
